# Supplementary material for: The relationship between perceptions of electronic health record usability and clinical importance of social and environmental determinants of health on provider documentation
Source: PLOS Digit Health. 2024 Jan 11;3(1):e0000428. doi: 10.1371/journal.pdig.0000428 (PMC10783763; doi:10.1371/journal.pdig.0000428)
Supplement: S1 Appendix — First 5 survey items assess basic demographic data, next 3 items assess SDH information (charting behavior, frequency of routine clinical consideration, EHR usability) and last 3 items assess EDH information (charting behavior, frequency of routine clinical consideration, EHR usability). (DOCX) [file pdig.0000428.s001.docx]

**S1 Appendix: Survey**

1. What is your role in the health system?
   1. Resident
   2. Fellow
   3. Attending, mostly clinical practice
   4. Attending, mostly research practice
   5. Attending, mostly administration
   6. Other
2. In what setting do you practice?
   1. Inpatient
   2. Outpatient
   3. Both inpatient and outpatient
3. In what department do you practice?
   1. Anesthesiology
   2. Dermatology
   3. Emergency medicine
   4. Family Medicine
   5. Internal Medicine and Subspecialties
   6. Neurology
   7. Obstetrics and Gynecology
   8. Ophthalmology
   9. Pathology
   10. Pediatrics
   11. Physical Medicine and Rehabilitation
   12. Psychiatry
   13. Radiology
   14. Radiation Oncology
   15. Surgery and Surgical Subspecialties
   16. Other
       1. Please specific your department: ____________________
4. What is your gender?
   1. Male
   2. Female
   3. Non-binary
   4. Another not listed
   5. Prefer not to answer
5. What is your ethnicity? Select all that apply.
   1. Hispanic/Latino
   2. Not Hispanic/Latino
   3. Prefer not to answer
6. Please rate how often you chart on the following social factors in your current practice.

*Never, Rarely, Sometimes, Often, Always*

- 1. Housing (housing age, mold, etc.)
  2. Food security (access to fresh fruits and vegetables)
  3. Transportation (work, medical)
  4. Utilities (electricity, air conditioning, heating)
  5. Childcare
  6. Employment (stable employment)
  7. Education
  8. Financial stability
  9. Personal safety (abuse, neighborhood safety)
  10. Access to guns in the household
  11. Insurance
  12. Smoking
  13. Migrant or Refugee Status

1. Please indicate your level of agreement with the following statement: “Routine consideration of the following social factors are vital in the clinical care of my patients.”

*Never, Rarely, Sometimes, Often, Always*

- 1. Housing (housing age, mold, etc.)
  2. Food security (access to fresh fruits and vegetables)
  3. Transportation (work, medical)
  4. Utilities (electricity, air conditioning, heating)
  5. Childcare
  6. Employment (stable employment)
  7. Education
  8. Financial stability
  9. Personal safety (abuse, neighborhood safety)
  10. Access to guns in the household
  11. Insurance
  12. Smoking
  13. Migrant or Refugee Status

1. The CERNER medical record system allows for me to easily document the following social factors.

*Yes, No*

- 1. Housing (housing age, mold, etc.)
  2. Food security (access to fresh fruits and vegetables)
  3. Transportation (work, medical)
  4. Utilities (electricity, air conditioning, heating)
  5. Childcare
  6. Employment (stable employment)
  7. Education
  8. Financial stability
  9. Personal safety (abuse, neighborhood safety)
  10. Access to guns in the household
  11. Insurance
  12. Smoking
  13. Migrant or Refugee Status

1. Please rate how often you chart on the following environmental factors in your current practice.

*Never, Rarely, Sometimes, Often, Always*

- 1. Air pollution
  2. Household pollution & toxicities
  3. Extreme weather (heat, cold)
  4. Access to basic appliances (e.g. electricity, air conditioning, heating)
  5. Exposures to natural disasters (e.g. hurricanes, wildfires, sea level rise, flooding)
  6. Infectious disease outbreaks
  7. Proximity to landfills, industrial centers, waste processing plants

1. Please indicate your level of agreement with the following statement: “Routine consideration of the following environmental factors are vital in my clinical care of patients.”

*Strongly disagree, Disagree, Neutral, Agree, Strongly agree*

- 1. Air pollution
  2. Household pollution & toxicities
  3. Extreme weather (heat, cold)
  4. Access to basic appliances (e.g. electricity, air conditioning, heating)
  5. Exposures to natural disasters (e.g. hurricanes, wildfires, sea level rise, flooding)
  6. Infectious disease outbreaks
  7. Proximity to landfills, industrial centers, waste processing plants

1. The CERNER medical record system allows for me to easily document the following.

*Yes, No*

- 1. Air pollution
  2. Household pollution & toxicities
  3. Extreme weather (heat, cold)
  4. Access to basic appliances (e.g. electricity, air conditioning, heating)
  5. Exposures to natural disasters (e.g. hurricanes, wildfires, sea level rise, flooding)
  6. Infectious disease outbreaks
  7. Proximity to landfills, industrial centers, waste processing plants
