# Supplementary material for: The relationship between perceptions of electronic health record usability and clinical importance of social and environmental determinants of health on provider documentation
Source: PLOS Digit Health. 2024 Jan 11;3(1):e0000428. doi: 10.1371/journal.pdig.0000428 (PMC10783763; doi:10.1371/journal.pdig.0000428)
Supplement: S1 Table — Table values correspond to the percentage of physicians by department who reported consistent charting on or vital to their practice. Only the SDHs with the highest percentage of physicians are listed. Multiple SDHs are listed if they had the same percentage of physicians reporting them. (DOCX) [file pdig.0000428.s004.docx]

**S1 Table**

**Top charted and clinically vital SDHs reported by department**

| Department | Top SDH charted | % | Top SDH perceived vital | % |
| --- | --- | --- | --- | --- |
| Anesthesiology | Smoking | 83 | Smoking | 100 |
| Dermatology | Smoking, Insurance | 33 | Employment | 100 |
| Family Medicine | Smoking | 92 | Smoking | 100 |
| Internal Medicine & Subspecialties | Smoking | 88 | Smoking | 88 |
| Neurology | Smoking | 100 | Smoking, Personal safety | 80 |
| Obstetrics & Gynecology | Smoking | 100 | Smoking | 100 |
| Pediatrics | Smoking | 57 | Personal safety | 76 |
| Psychiatry | Education | 100 | Smoking, Childcare, Employment, Personal safety,  Access to guns | 100 |
| Surgery & Surgical Subspecialties | Smoking | 85 | Smoking | 93 |
| Emergency Medicine | Smoking | 83 | Housing,  Personal safety | 100 |
| Pathology | Smoking | 100 | Smoking | 100 |
| Physical Medicine & Rehabilitation | Smoking | 100 | Smoking,  Transportation | 100 |
| Radiology | Smoking,  Transportation | 17 | Smoking | 83 |
| Other | Smoking | 83 | Transportation, Employment, finance | 83 |

Table values correspond to the percentage of physicians by department who reported consistent charting on or vital to their practice. Only the SDHs with the highest percentage of physicians are listed. Multiple SDHs are listed if they had the same percentage of physicians reporting them.

**Supplemental Table 5. Top charted and clinically vital EDHs reported by department**

| Department | Top EDH charted | % | Top EDH perceived vital | % |
| --- | --- | --- | --- | --- |
| Anesthesiology | Infectious disease | 33 | Infectious disease, Natural disasters | 83 |
| Dermatology | None reported | 0 | Infectious disease | 100 |
| Family Medicine | Infectious disease | 38 | Infectious disease | 88 |
| Internal Medicine & Subspecialties | Infectious disease | 18 | Infectious disease | 88 |
| Neurology | Infectious disease | 40 | Infectious disease,  Extreme weather | 80 |
| Obstetrics & Gynecology | Infectious disease | 45 | Infectious disease | 100 |
| Pediatrics | Infectious disease | 33 | Infectious disease | 76 |
| Psychiatry | Infectious disease | 55 | Infectious disease | 90 |
| Surgery & Surgical Subspecialties | Infectious disease | 19 | Infectious disease | 67 |
| Emergency Medicine | Infectious disease | 50 | Infectious disease | 83 |
| Pathology | Infectious disease | 100 | Infectious disease, Household pollution,  Proximity to landfills | 100 |
| Physical Medicine & Rehabilitation | Infectious disease | 25 | Infectious disease, Natural disasters | 75 |
| Radiology | Infectious disease | 33 | Infectious disease | 50 |
| Other | Infectious disease | 33 | Access basic appliances | 67 |

Table values correspond to the percentage of physicians by department who reported consistent charting on or vital to their practice. Only the EDHs with the highest percentage of physicians are listed. Multiple EDHs are listed if they had the same percentage of physicians reporting them.
