# Supplementary material for: The relationship between perceptions of electronic health record usability and clinical importance of social and environmental determinants of health on provider documentation
Source: PLOS Digit Health. 2024 Jan 11;3(1):e0000428. doi: 10.1371/journal.pdig.0000428 (PMC10783763; doi:10.1371/journal.pdig.0000428)
Supplement: Attachment — Submitted filename: Article Review-PDIG-D-23-00183_R1.pdf [file pdig.0000428.s009.pdf]

The relationship between perceptions of electronic health record usability and clinical importance of social and environmental determinants of health on provider documentation--- Review

A. **SUMMARY OF THE RESEARCH FINDINGS AND OVERALL IMPRESSION**
   i. **Summary of the Research findings**

Abstract section

The authors have provided a good overview of the Social and environmental determinants of health, and electronic health records. The focus of the study is clearly provided. The data collection method use is mentioned. The total providers who participated have been provided, but it is not clear from what pool of target participants they were sampled and what sampling technique was used. Also the methodology used in the study is not mentioned. The authors have provided an insightful results summary as well as the area for further research.

The authors found out some relationships, **the question here is so what**? Some recommendations from the relationships found should be drawn so as to support either policy or better implementation or clinical practice. Otherwise, finding the relationship may be trivial but generating sense and guidance for a way forward adds more value and would be a good contribution. Refer to line 366 to generate this information.

Since the abstract is a central place for reviewing the whole article, a summary of all the information is important to enable the readers have a one-time view of the article before digging down to the details.

Other sections

Feedback on the reviewed comments and responses in their order as stated in the article PDIG-D-23-00183_R1

Reviewed comment 1

The study team acknowledges this comment and appreciates the feedback. Substantial edits have been made to the introduction to explain determinants of health and what is considered SDH vs EDH and SEDH for the purposes of the study to provide more clarity for the reader.

Feedback

These have been incorporated and it is now clear. Evident on..line 72, 97-109

Reviewed comment 2

The study team appreciates this comment. Given the significant edits made to the introduction (as stated above), we believe this issue has been addressed. We invite the reviewer to verify that the edits already made to the introduction have addressed this issue.

Feedback

Clarification has been made concerning the SDH and EDH and SEDH and now the correlation with the objectives achievement is quite clearer.

Reviewed comment 3

Edits to this section include further information as to how the study population was identified. Specifically, all staff physicians were able to participate in this study and were identified using real-time administrative listservs though the Medical Staff Office (to identify attending physicians) and the Graduate Medical Education Office (to identify physicians in training - residents and fellows). All staff physicians (study population) were sent an email with the link to the REDCap survey and had the opportunity to voluntarily complete the survey. Given the total possible population of 2,478 staff physicians at our institution, the study team deemed that it was not necessary to select a smaller sample for this study because it was feasible to analyze all 2,478 responses if we were able to achieve a 100% response rate. One objective of this study was to obtain a general baseline incidence of SEDH charting behavior and SEDH clinical consideration behavior and to the best knowledge of the study team, the incidences of these behaviors (as related to each specific SEDH element) in physicians (attendings, residents and fellows) is not yet currently known and was not available to use for power calculation or sample size calculation prior to our study.

Feedback

Just like a comment I made under the abstract. This was a relevant review comment. The methodology/study design needs to appear in the abstract as well as other components as commented above

The authors have incorporated the study design Information, and how participants were recruited. Also mentioned that the Institution approval was obtained but usually its good practice to provide the approval number as evidence.

A total of 2,478 staff physicians is talked about and a sample of 201 participants that completed the survey is mentioned. However until now the sampling technique used is not mentioned. For example, was it something like Krejcie & Morgan (1970) tables, or something? This is still missing.

Reviewed comment 4

Edits to this section have been made to state, “A total of 2,478 staff physicians (1,800 attending physicians and 678 residents/fellows) were identified using an administrative listserv at the study institution during the project timeframe. Of this total, 201 participants completed the survey (8.1% response rate) and were included in the analysis.”

Feedback

The information was incorporated but as mentioned above the sampling technique is missing, this is a critical data collection, management and analysis aspect that cannot miss.

Reviewed comment 5

The study team acknowledges the reviewer comment and invite the reviewer to see the above edits that have been made in the results section to address this issue

Comment

There are some explanations put but none is showing a clear working or formula that was used to generate 8%. The authors can provide the same for clarity to the reviewer’s comments.

ii. **Overall impression.**

The authors have put in efforts to provide clarity on a number of aspects. Yes the article has greatly improved BUT some few areas are not yet fully clarified. These are few **BUT CRITICAL** and can be rectified in the shortest time possible by the authors and possibly the article accepted for publication.

B. SPECIFIC AREAS OF IMPROVEMENT

- ✓ Add missing information in the abstract as indicated in the abstract comments section
- ✓ Provide an answer to the so what question asked, include it in the abstract as a critical recommendation. Part of this information is actually in the conclusion section line 366 starting with "improving…"
- ✓ Add ethical approval number
- ✓ Provide sampling technique used
- ✓ Provide the formulae (or in nutshell the calculation) used to arrive at 8% response rate.
