# Supplementary material for: The relationship between perceptions of electronic health record usability and clinical importance of social and environmental determinants of health on provider documentation
Source: PLOS Digit Health. 2024 Jan 11;3(1):e0000428. doi: 10.1371/journal.pdig.0000428 (PMC10783763; doi:10.1371/journal.pdig.0000428)
Supplement: Attachment — Submitted filename: commnets to Authors_ PDIG-D-23_00183.docx [file pdig.0000428.s006.docx]

**Comments to authors**

General comments. Overall structure of the manuscript should include sub themes describing the processes carried out during the study. For example under material and methods section, data collection, sampling process and study design has been boxed together. There are component under the study design which should be moved to the introduction section to bring clarity to the stated acronyms

**Introduction**

What are some of the elements of SEDH? Can the authors be clear on what they define as SEDH for readers to appreciate what to expect? Also can the authors clarify what EDH is ? The introduction is not clear on how the providers come to be associated with SEDH and EDH. The manuscript can be improved if the authors state and clarify these issues early. As it stands now, the understanding and interpretation of SEDH and EDH are to the discretion of the reader.

Although the Objectives of the study are stated clearly, the lack of clarity of SEDH, EDH and how providers are associated with it at the onset, obscures how the stated objectives can be achieved.

**Methods**

Under study design, this should state clearly the study type, when and where it was conducted. The text describing the data collection tools should be captured separately under different subtheme to bring clarity. Again, there should be a section on how data was collected, and the procedures involved to aid understanding how the study was carried out

Again the part of text under the survey design which defines SEDH, EDH SDH should be moved under the introduction

How were participants selected or recruited into the study? How was sampling and sample size determined?

**Results**

Line 173-174 the authors states: “ The survey was administered to all physicians at a single academic health center, of which a total of 201 completed the survey…” How many physicians were in this facility? What was the response rate?

**Discussion**

Again on Line 353 the authors’ state “We had a low overall response rate of 8% which…” How did the authors arrived at this figure?
