# Supplementary material for: The relationship between perceptions of electronic health record usability and clinical importance of social and environmental determinants of health on provider documentation
Source: PLOS Digit Health. 2024 Jan 11;3(1):e0000428. doi: 10.1371/journal.pdig.0000428 (PMC10783763; doi:10.1371/journal.pdig.0000428)
Supplement: Attachment — Submitted filename: Response to Reviewers.docx [file pdig.0000428.s007.docx]

**Ref: Manuscript Number PDIG-D-23-00183**

**Title: The relationship between perceptions of electronic health record usability and clinical importance of social and environmental determinants of health on provider documentation**

Dear Editor in Chief

Thank you for the thorough and constructive review of our manuscript. We have included both a marked-up copy with TrackChanges as well as an unmarked version as per instructions. Please see below for an itemized response to each reviewer comment,

We hope that the revisions made have addressed the reviewers’ concerns about the manuscript. We believe that these revisions have greatly enhanced our manuscript. We are grateful for the opportunity to resubmit the manuscript. Please feel free to contact me should you have further questions.

Sincerely,

Natasha Sood, MD, MPH

Pennsylvania State College of Medicine

700 HMC Cres Rd.

Hershey, PA 17033

**Comments to authors**

General comments. Overall structure of the manuscript should include sub themes describing the processes carried out during the study. For example under material and methods section, data collection, sampling process and study design has been boxed together. There are component under the study design which should be moved to the introduction section to bring clarity to the stated acronyms

**Introduction**

What are some of the elements of SEDH? Can the authors be clear on what they define as SEDH for readers to appreciate what to expect? Also can the authors clarify what EDH is ? The introduction is not clear on how the providers come to be associated with SEDH and EDH. The manuscript can be improved if the authors state and clarify these issues early. As it stands now, the understanding and interpretation of SEDH and EDH are to the discretion of the reader.

The study team acknowledges this comment and appreciates the feedback. Substantial edits have been made to the introduction to explain determinants of health and what is considered SDH vs EDH and SEDH for the purposes of the study to provide more clarity for the reader.

Although the Objectives of the study are stated clearly, the lack of clarity of SEDH, EDH and how providers are associated with it at the onset, obscures how the stated objectives can be achieved.

The study team appreciates this comment. Given the significant edits made to the introduction (as stated above), we believe this issue has been addressed. We invite the reviewer to verify that the edits already made to the introduction have addressed this issue.

**Methods**

Under study design, this should state clearly the study type, when and where it was conducted. The text describing the data collection tools should be captured separately under different subtheme to bring clarity. Again, there should be a section on how data was collected, and the procedures involved to aid understanding how the study was carried out

The study team appreciates this reviewer comment for further clarification. Edits to this section include the following: inclusion of the subsections “study design,” “data collection tool,” and “statistical analysis.” Each subsection provides further clarity on the study procedures, survey description and analysis. The “ethics statement” subsection was also moved to the end of the Methods section for improved flow of reading. Specifically related to this comment, the study type of “cross-sectional study” conducted at “Penn State Health Milton Hershey Medical Center” from “September to October 2021” has been included under the “study design” section. This section also specifies in detail how the survey was distributed to all identified “staff physicians” at the study institution electronically by using institutional email addresses through the administrative offices of the Medical Staff and Graduate Medical Education Offices.

Again the part of text under the survey design which defines SEDH, EDH SDH should be moved under the introduction

This portion of text defining SEDH/SDH/EDH has been moved up under the introduction to provide clarity for the reader.

How were participants selected or recruited into the study? How was sampling and sample size determined?

Edits to this section include further information as to how the study population was identified. Specifically, all staff physicians were able to participate in this study and were identified using real-time administrative listservs though the Medical Staff Office (to identify attending physicians) and the Graduate Medical Education Office (to identify physicians in training - residents and fellows). All staff physicians (study population) were sent an email with the link to the REDCap survey and had the opportunity to voluntarily complete the survey. Given the total possible population of 2,478 staff physicians at our institution, the study team deemed that it was not necessary to select a smaller sample for this study because it was feasible to analyze all 2,478 responses if we were able to achieve a 100% response rate. One objective of this study was to obtain a general baseline incidence of SEDH charting behavior and SEDH clinical consideration behavior and to the best knowledge of the study team, the incidences of these behaviors (as related to each specific SEDH element) in physicians (attendings, residents and fellows) is not yet currently known and was not available to use for power calculation or sample size calculation prior to our study.

**Results**

Line 173-174 the authors states: “ The survey was administered to all physicians at a single academic health center, of which a total of 201 completed the survey…” How many physicians were in this facility? What was the response rate?

Edits to this section have been made to state, “A total of 2,478 staff physicians (1,800 attending physicians and 678 residents/fellows) were identified using an administrative listserv at the study institution during the project timeframe. Of this total, 201 participants completed the survey (8.1% response rate) and were included in the analysis.”

**Discussion**

Again on Line 353 the authors’ state “We had a low overall response rate of 8% which…” How did the authors arrived at this figure?

The study team acknowledges the reviewer comment and invite the reviewer to see the above edits that have been made in the results section to address this issue.
