# Supplementary material for: The relationship between perceptions of electronic health record usability and clinical importance of social and environmental determinants of health on provider documentation
Source: PLOS Digit Health. 2024 Jan 11;3(1):e0000428. doi: 10.1371/journal.pdig.0000428 (PMC10783763; doi:10.1371/journal.pdig.0000428)
Supplement: Attachment — Submitted filename: comments to authors R1 PDIG-D-23_00183.docx [file pdig.0000428.s008.docx]

General comments

The authors have addressed all my concerns. The manuscript has greatly improved.

Minor comment

Sentences on Lines 132-133 should either be deleted or moved to the subtheme: Ethics statement
