# Supplementary material for: The relationship between perceptions of electronic health record usability and clinical importance of social and environmental determinants of health on provider documentation
Source: PLOS Digit Health. 2024 Jan 11;3(1):e0000428. doi: 10.1371/journal.pdig.0000428 (PMC10783763; doi:10.1371/journal.pdig.0000428)
Supplement: Attachment — Submitted filename: Response to Reviewers.docx [file pdig.0000428.s010.docx]

**Ref: Manuscript Number PDIG-D-23-00183**

**Title: The relationship between perceptions of electronic health record usability and clinical importance of social and environmental determinants of health on provider documentation**

Dear Editor in Chief

Thank you for the thorough and constructive review of our manuscript. We have included both a marked-up copy with TrackChanges as well as an unmarked version as per instructions. Please see below for an itemized response to each reviewer comment.

We appreciate the reviewers’ detail to further optimize this revised draft. We appreciate the comment about further detailing the associations that we found in this study. We believe that these revisions have greatly enhanced our manuscript. We are grateful for the opportunity to resubmit the manuscript. Please feel free to contact me should you have further questions.

Sincerely,

Sona Jasani, MD

Yale New Haven Hospital

333 Cedar Street

New Haven, Connecticut 06510

____________________________________________________________________________

General comments

The authors have addressed all my concerns. The manuscript has greatly improved.

Minor comment

Sentences on Lines 132-133 should either be deleted or moved to the subtheme: Ethics statement

Response
Sentences on Lines 132-133 were moved and incorporated within lines 161-163 under Ethics Statement.

**B. SPECIFIC AREAS OF IMPROVEMENT**

Add missing information in the abstract as indicated in the abstract comments section

Response
The authors appreciate this comment to improve the abstract section. The following edits have been made:
1. Clearly describe the pool of target participants (see lines 35-37)
The study team has adjusted this section to state that the target pool of participants included all staff physicians identified through administrative listserves. We have also clearly stated the total identified pool of participants (2,478) and the number of physicians who responded/completed the survey (201) for a calculated response rate of (8.1%) 🡪 [ {201 divided by 2478} x 100 = 8.1% ]. There was not an additional sampling method used as the survey was sent to all identified staff physicians (the study did not further “select” from this identified population of staff physicians). The study team feels that this additional clarity addresses the reviewer’s comment and appreciate additional specific details regarding the reviewer’s concerns if additional questions still exist.

2. Clearly described the study design and methodology (see lines 32-33 and lines 37-44)
The study design has been described with the following addition/edits: “We performed a cross-sectional study using an 11-question electronic survey to assess self-reported practices related to clinical consideration of SEDH elements, EHR usability and SEDH documentation…”. The methodology regarding analysis has also been included in a brief description for more clarity as indicated by the reviewer’s comments: “A five-point Likert scale from “never” to “always” assessed charting behavior and clinical consideration. Responses were dichotomized as consistent/inconsistent and vital/not vital respectively. EHR usability was assessed as “yes” or “no” responses. Fisher’s exact tests assessed the relationship between charting behavior and clinical consideration and to compare charting practices between different SEDHs. Cumulative measures were constructed for consistent charting and ease of charting. A generalized linear mixed model (GLMM) compared SDH and EDH with respect to each cumulative measure and was quantified using odds ratios (OR) and 95% confidence intervals (CI).”

3. Address the question “so what” (see lines 44-56)
The following edits have been made to state this in the abstract section. “Our results show that provider documentation frequency of an SEDH is associated with perceived clinical utility as well as ease of charting and that providers were more likely to consistently chart on SDH versus EDH. Nuances in these relationships did exist with one notable example comparing the results of smoking (SDH) to infectious disease outbreaks (EDH). Despite similar percentages of physicians reporting that both smoking and infectious disease outbreaks are vital to care, differences in charting consistency and ease of charting between these two were seen. Taken as a whole, our results suggest that SEDH quality optimization efforts cannot consider physician perceptions and EHR usability as siloed entities and that EHR design should not be the only target for intervention. The associations found in this study provide a starting point to understand the complexity in how clinical utility and EHR usability influence charting consistency, however, further research is needed to understand how these relationships intersect at various levels in the SEDH data optimization process.”

Provide sampling technique used/Provide the formulae (or in nutshell the calculation) used to arrive at 8% response rate.
Response
Please see #1 in the above response to the requested edits to the abstract.

Provide an answer to the so what question asked, include it in the abstract as a critical recommendation. Part of this information is actually in the conclusion section line 366 starting with “improving”

Response
The authors appreciate this comment to strengthen the manuscript in answering the “so what question.” Please refer to lines 400-416 to see edits to the conclusion to flush out this idea and provide some clear guidance on how to approach improving SEDH data quality based on the take home point. The included edit reads as follows, “In conclusion, our study results show that provider documentation frequency of an SEDH is associated with clinical utility as well as ease of charting and that providers were more likely to consistently chart on SDH versus EDH. There is nuance, however, in these associations. Though a comparable percentage of physicians reported that smoking (SDH) and infectious disease outbreaks (EDH) were vital to care, there were still notable differences in charting consistency and ease of charting between these two. Inconsistent and incomplete acquisition affects the quality and use of SEDH data so understanding the factors that promote or hinder provider capture and entry should be a central consideration in any optimization effort. These efforts however cannot only focus on EHR design or physician perception as siloed entities, as suggested from our results. Instead, it may be more beneficial to approach data quality optimization using a multi-level framework: up-stream factors (examining education and training curricula), care delivery factors (establishing guidelines to systematize collection and storage) and down-stream factors (providing feedback through metrics on clinical use of SEDH elements and documentation practices). Our results provide a starting point to understand how clinical utility and EHR usability influence physician documentation practices for each individual SEDH element. Further research is still needed to explicate these associations at each of these three levels as well as to identify sustainable strategies to improve SEDH data quality and utility.”

Add ethical approval number
Response
The authors would like to direct the reviewer to line 195 regarding the ethical approval number which was already included in the previous manuscript as well as this revised copy “STUDY00018033.” The study team appreciates additional details regarding this reviewer concern if additional questions exist.
